# A focus on sustainable method development for greener synthesis

**DOI:** 10.1039/d3sc90120c

**Published:** 2023-06-29

**Authors:** Jasper L. Tyler, Felix Katzenburg, Frank Glorius

**Affiliations:** a Westfälische Wilhelms-Universität Münster, Organisch-Chemisches Institut Corrensstraße 36 48149 Munster Germany glorius@uni-muenster.de

## Abstract

Given the current global climate and health challenges, sustainability and cost-effectiveness are becoming unavoidable factors that must be considered in the development of new synthetic methodologies. In a recent publication, Kavthe *et al.* (R. D. Kavthe, K. S. Iyer, J. C. Caravez and B. H. Lipshutz, *Chem. Sci.*, 2023, **14**, 6399, https://doi.org/10.1039/D3SC01699D) have succinctly demonstrated how employing more sustainable methodology can vastly reduce the environmental impact associated with the synthesis of the antimalarial drug candidate MMV688533. The most notable feature of this newly reported synthetic route is the application of aqueous micellar conditions to two Sonogashira coupling reactions that simultaneously improve the yield, catalyst loading and sustainability of these key steps.

## Abstract 2

Kavthe *et al.* (*Chem. Sci.*, 2023, https://doi.org/10.1039/D3SC01699D) have demonstrated how employing more sustainable methodology can vastly reduce the environmental impact associated with the synthesis of the antimalarial drug candidate MMV688533.

## Graphical abstract

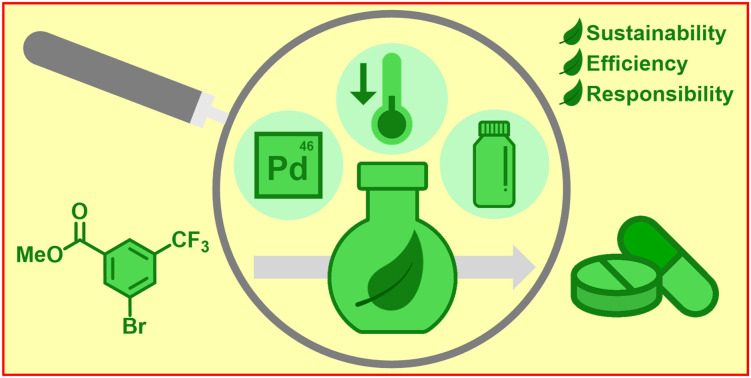

Innovative and powerful synthetic methods are the enabling technologies that allow access to active pharmaceutical ingredients (APIs) and given the global climate and health challenges, sustainability and cost-effectiveness are becoming increasingly important in method development. Expanding from a reductionist view on methodologies focused on product structures and yields, a more holistic approach considering overall environmental impact and cost sets a path to access and mass-produce compounds of significant importance, such as drugs.

The development and manufacturing of drugs are key to promoting global health. Considering the existing epidemics of AIDS, tuberculosis and malaria, as well as the emergence of resistant pathogens, effective treatments that directly address the United Nations 3rd Sustainable Development Goal (SDG) “Good Health and Well-Being” (Fig. 1A) are of paramount importance.^1^ However, a solution to this challenge should avoid conflicting with other sustainability challenges. The manufacturing of pharmaceuticals typically generates waste that exceeds the mass of the generated product by 25 to 100 times.^2^ But besides reducing environmental impact, efficient synthetic routes and less hazardous processes are also crucial for the cost-effective production of APIs that are needed globally. These connected challenges set efficient API synthesis in the context of the 10th (“Reduced Inequalities”) and 12th (“Responsible Consumption and Production”) SDGs and highlight the need for so-called “nexus solutions” that synergistically address multiple sustainability challenges.^3^

**Fig. 1:**
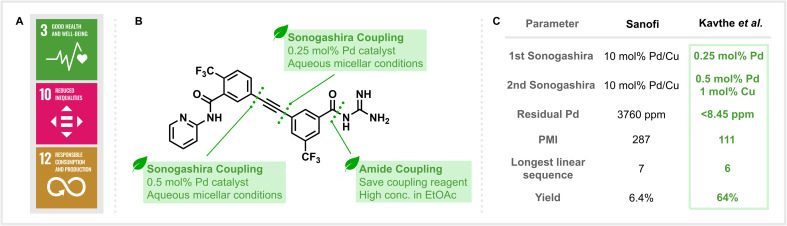
(A) The United Nations Sustainable Development Goals related to sustainable drug manufacturing. (B) Key retrosynthetic disconnections in the sustainable synthesis of MMV688533. (C) Comparison of key parameters in the syntheses reported by Kavthe *et al.*^10^ and Sanofi.^12^

In this regard, sustainable method development should ideally seek solutions that combine several desirable design paradigms. Three frequently highlighted challenges: (i) efficient catalysis, (ii) sustainable solvents and (iii) mild reaction conditions can serve as guiding points when developing and accessing new methods.

Catalysis plays a central role in the development of efficient and selective reactions. Due to the frequent use of precious metals in catalytic systems and the strict requirements for residual metal levels in drug formulations, the reduction of catalyst loadings and immobilisation of catalysts are two approaches with the potential to render precious metal catalysis more sustainable. The development of sub-ppm level asymmetric organocatalysis and first-row transition metal couplings show further progress towards more efficient catalytic systems.^4^

Solvents can contribute up to 85% of the total raw material mass that is used for API synthesis.^5^ Therefore, selecting and optimising greener solvents based on resources like the CHEM21 selection guide can contribute significantly to the overall sustainability of a reaction.^6^ The use of higher concentrations or eliminating the need for solvents under neat or mechanochemical conditions are also promising alternatives.

More sustainable reaction conditions are often collectively referred to as “mild” in method development.^7^ While being loosely defined, the term reflects efforts to integrate more energy-efficient and less hazardous conditions as a design decision. The recent report of a visible-light-driven variant of the Birch reduction is a prime example of how milder reaction conditions can eliminate the need for cryogenic temperatures and the use of toxic, environmentally hazardous and pyrophoric reagents.^8^

Efforts to meet these challenges are being driven by vital discoveries from both the chemical industry and numerous research groups. In this regard, the Lipshutz laboratory has been at the forefront of developing new methodologies in the field of green and environmentally responsible chemistry, pioneering the use of water as a solvent for organic reactions which have been historically considered to be incompatible with this medium. The realisation of this move to greener solvents has been facilitated by the group’s design and application of non-ionic surfactants that spontaneously self-aggregate in water to form micelles. These assemblies have been repeatedly demonstrated to be capable of acting as “nanoreactors” in which highly efficient catalytic processes can be performed, enabling significant reductions in loadings of precious and often toxic transition metal catalysts.^9^

In their most recent publication (Kavthe *et al.*, https://doi.org/10.1039/D3SC01699D),^10^ the methods and principles described above were directly applied to the development of an alternative synthetic route to MMV688533 (Fig. 1B), a promising new single-dose malaria treatment with a high barrier to *Plasmodium falciparum* parasite resistance which is currently in the advanced stages of clinical evaluation.^11^ Not only was a practical, robust, and environmentally responsible synthesis targeted, but attention was also given to the cost-effectiveness of the newly developed route. This factor is particularly important to the large-scale production of drugs, such as antimalarials, which will be almost exclusively distributed in the developing world.

From an environmental viewpoint, the issues associated with the original discovery route reported by Sanofi are numerous.^12^ The process, with an overall yield of 6.4% based on the longest linear sequence, is step-inefficient and utilises two Sonogashira coupling reactions that require high loadings of palladium catalyst (10 mol%). The use of traditional amide coupling reagents (such as DCC) also leads to the generation of stoichiometric waste. Lastly, all reactions are carried out in organic and, in part, health and environmentally hazardous solvents.

The application of the group’s self-assembling surfactant methodology to both Sonogashira cross-coupling reactions was highly successful and allowed these key C–C bond forming reactions to be performed in aqueous media, albeit with 10% (v/v) THF as a co-solvent. Notably, the change of conditions could also be accompanied by a significant drop in the amount of metal catalyst required. This manifested itself as a 20-fold decrease in the loading of Pd and a 10-fold decrease in the loading of Cu for the coupling reaction performed at the later stage (Fig. 1C). Moreover, for the earlier Sonogashira reaction, no Cu co-catalyst and only 2500 ppm of Pd were needed to provide the corresponding product in 96% yield. The reduction in the loading of palladium catalyst is not only beneficial due to the reduction in cost associated with employing a precious metal with a finite natural supply but also has a positive downstream effect. Kavthe *et al.* employed ICP-MS analysis of the newly synthesised MMV688533 to reveal that only <8.45 ppm of residual palladium was present, a level that lies under the FDA-allowed limit of 10 ppm per day per dose and represents a significant reduction from the 3760 ppm found in the sample prepared according to the previous literature method. Additionally, a key amide bond forming reaction was achieved using the group’s recently reported thioester-based protocol that eliminates the need to use traditional amide coupling reagents (such as DCC) and generates easily removable and recyclable by-products.^13^

Ultimately, redesigning the route led to a significantly improved efficiency in the synthesis of MMV688533, with a six-step longest linear sequence providing an overall yield of 64% (a 10-fold increase compared to the initial method). Perhaps the most important difference is that all of the transformations for the synthesis in question can be performed under neat, green solvent or aqueous micellar conditions. Quantitatively assessing the production of waste in both syntheses found that the process mass intensity (PMI) of the newly reported route (111 kg input per kg product) is less than half of what was calculated for the discovery route (287 kg input per kg product), highlighting the level of improvement in sustainability that this work has achieved.^14^

Overall, the synthesis of MMV688533 presented by Kavthe *et al.*^10^ demonstrates how environmentally orientated methods can translate to more sustainable synthesis. By developing a new synthetic route featuring Sonogashira couplings under aqueous micellar conditions: PMI, catalyst loading, step economy and yield could all be simultaneously improved. Despite this, there is still room for further improvements, such as avoiding organic co-solvents and the use of stoichiometric activation reagents. With respect to industrial applicability, previous applications of this surfactant technology on-scale have already demonstrated that modern lab-scale method development can indeed translate to process chemistry.^15^

The diverse challenges involved in establishing new and improving existing chemical processes will continue to require the development of novel synthetic methods. In an increasingly data-driven world, the standardisation of data reported in scientific publications is essential to allow a direct comparison of the sustainability of laboratory reactions and help guide decisions with respect to the transfer of new protocols to large-scale synthesis.^16^ In conjunction with this, improved metrics which quantify the overall environmental impact of a reaction and consider a broader scope of sustainability factors are urgently needed. These efforts, together with research targeting the inclusion of renewable feedstocks and the recycling of valuable materials will help chemistry reach its central role in setting a path for a more sustainable future.

## Author contributions

J. L. T., F. K. and F. G. wrote the manuscript.

## Conflicts of interest

There are no conflicts to declare.

## Supplementary Material
